# Synthetic Data-Driven Exoskeleton Control via Contralateral Gait Fusion for Variable-Speed Walking

**DOI:** 10.3390/biomimetics11050319

**Published:** 2026-05-03

**Authors:** Jingshu Shi, Hongwu Zhu, Yifei Yang, Bowen Liu, Xingjun Wang

**Affiliations:** 1 Shenzhen International Graduate School, Tsinghua University, Shenzhen 518055, China; shi-js23@mails.tsinghua.edu.cn (J.S.); yangyife24@mails.tsinghua.edu.cn (Y.Y.); 2 Innovation Department Skyworth Digital Co., Ltd., Shenzhen 518000, China; fuquph@gmail.com

**Keywords:** data-driven, exoskeleton, reinforcement learning, sim-to-real transfer, contralateral gait

## Abstract

Data-driven exoskeletons offer the potential for adaptive augmentation of human mobility. Yet their widespread adoption is hindered by labor-intensive biomechanical data collection and manual tuning. Herein, this study presents a highly efficient synthetic data approach to facilitate data-driven pipelines. We leveraged an Adversarial Motion Priors (AMP) agent to learn stylized walking within a massively parallel, physics-based simulation. The resulting high-fidelity data were collected and validated against OpenSim inverse dynamics pipelines. Further, we trained an end-to-end torque prediction algorithm using the collected data. A novel CNN-Transformer architecture was developed to map contralateral swing-phase data to variable-length push-off torque profiles. This enabled real-time, adaptive torque assistance of exoskeletons for variable-speed walking. A custom ankle exoskeleton was used to demonstrate robust sim-to-real transferability. Our system achieved an average root mean square error of approximately 0.081 ± 0.015 newton-meters per kilogram and an average R^2^ of 0.836 ± 0.050 across speeds ranging from 0.6 to 1.75 m·s^−1^. The controller significantly reduced user-positive ankle mechanical work by up to 14 ± 6.30%. Finally, our multi-sensor configuration exhibited inherent fault tolerance, ensuring safe operation even under partial sensor failure. By taking a scalable, data-driven approach, this work offers a practical pathway toward deploying autonomous exoskeletons in versatile, real-world environments.

## 1. Introduction

Powered exoskeletons hold transformative potential for augmenting human mobility [1,2,3,4]. Yet their widespread adoption is stifled by the rigidity of conventional control architectures. Dominant hierarchical approaches rely on high-level discrete task classification to trigger mid-level phase-based spline torque profiles [5,6,7,8]. Such methods inherently need labor-intensive handcrafted controllers for unstructured, non-cyclic, or transitory motions in daily life. Some automatic optimizations of control parameters were available for specific tasks, requiring participants to undertake experimental trials or metabolic cost measurements [9,10]. These task-specific controllers fail to achieve generalization. This creates an urgent need for a versatile strategy that operates without explicit task classification or exhaustive manual tuning.

Classifiers have been adopted to enable autonomous mode transitions between assistive strategies. For high-level control, data-driven models can estimate more high-level states compared with physics-driven models classifier [11]. A data-driven model can continuously classify the environment and estimate the phase, phase rate, and stride length during locomotion [12,13]. Computer vision and deep learning were leveraged to classify environments and estimate terrains in both indoor and outdoor settings outdoors [14,15]. Proprioceptive sensors provide kinematic data for neural networks. These networks detect stance/swing transitions and provide proper assistance at the desired timing during variable-speed walking [11,16]. For mid-level control, human-in-the-loop optimization utilized respirometry measurement as the metabolic cost. Control law optimization could be solved online via repeated trials [2]. When the above cost is replaced with a data-driven classifier, the optimization process can be performed rapidly in real-world conditions [17]. Meanwhile, some end-to-end data-driven methods demonstrated superior robustness across diverse individuals and environments [18,19]. These models leverage continuous physiological states as input and biological joint moments as output to enable real-time torque estimation. However, despite their superiority in flexibility, such end-to-end data-driven approaches were “data-hungry,” requiring large quantities of training data.

To collect biomechanical ground-truth labels, users must perform repeated diverse tasks in laboratories equipped with motion capture (kinematics) and force plates (ground reaction forces). Subsequently, musculoskeletal-based OpenSim inverse dynamics were applied to calculate joint moments [20,21,22]. Given the labor-intensive and complex nature of this process, researchers also open-sourced several online datasets [19,23,24]. Meanwhile, simulation-based synthetic data generated by generative models presented a promising alternative paradigm. This paradigm has been firmly established in robotic locomotion and manipulation [25,26]. Accurate latent dynamics could be captured by data-driven reinforcement learning (RL) for locomotion control [27]. Relying on reward shaping and domain randomization, the trained policy could overcome the sim2real gaps [28,29,30,31]. Imitation learning, such as generative adversarial imitation learning (GAIL) and discriminator-actor-critic (DAC), could duplicate reference motions [32,33], but lacked physical grounding. Adversarial motion priors (AMPs) had both dynamics-aware attributes and duplicate ability, which were widely used in humanoid robotics. It utilized an adversarial motion prior to learning the distribution of reference motions. This ensured that the generated policy was not only stylistically realistic but also physically plausible, which could serve as a powerful tool for data generation and validation [34].

Inspired by this concept, we trained a stylized walking agent in simulation to generate massive synthetic datasets with precise labels. We obtained graceful and life-like walking behaviors from the AMP agent [35,36,37], and then collected both sensor readings and torque profiles. A rigorous validation of the walking agent against the OpenSim standard pipeline was made. The generated torque curves closely match the standard experimental results with high consistency, indicating that the agent successfully learns walking dynamics. Subsequently, we proposed a novel CNN-Transformer algorithm. Convolutional Neural Networks (CNNs) extract robust local features, while transformers capture long-range temporal dependencies. Together, they enabled the precise mapping of torque profiles. This algorithm was implemented on an ankle exoskeleton. The system ensured adaptability across walking speeds from 0.6 m·s^−1^ to 1.75 m·s^−1^ at a torque average coefficient of determination of 0.836 ± 0.050 and an average root mean square error of approximately 0.081 ± 0.015 newton-meters per kilogram. We demonstrated that this task-agnostic controller significantly reduced user-positive ankle mechanical work by up to 14 ± 6.30%, marking a critical step in exoskeleton technologies.

## 2. Materials and Methods

### 2.1. The Simulation Environment and Exoskeleton Hardware Architecture

All simulations were executed on a workstation equipped with an Nvidia RTX 6000 Ada Generation GPU (Driver version: 580.126.09, CUDA v12.9.41). The simulation framework utilized Isaac Sim (v4.5.0) integrated with PyTorch (v2.5.1+cu118) for deep learning training and validation. Biomechanical analyses were conducted via OpenSim (v4.5). To acquire ground-truth kinetic data, we employed an optical motion capture (NOKOV, Mars 1.3H) synchronized with high-fidelity force plates (Bertec, FP4060).

The custom power augmentation ankle exoskeleton platform comprised one active dorsiflexion/plantarflexion joint and one passive inversion/eversion joint for each leg, driven by a Xiaomi CyberGear motor (Xiaomi Corp., Beijing, China, gear ratio 7.75:1). The embedded controller (RDK X5, d-robotics, Shenzhen, China) operated at a frequency of 500 Hz to ensure real-time responsiveness. Proprioceptive sensing for each leg was achieved through a multi-sensor configuration: 6-axis inertial measurement unit (IMU) (YIS320, 200 Hz, Yesense, Wuhan, China), 1-channel encoder (eCoder 11, 1000 Hz, ZeroErr, Shenzhen, China), and 1-channel 940 nm laser time-of-flight (ToF) sensor (TOFSense-F/F2, 50 Hz, NooPLoop, Shenzhen, China). All sensors were mounted on the shank of the exoskeleton. Sensor data were resampled to 500 Hz using spline interpolation. IMU signals were preprocessed with a 5 Hz low-pass filter. The dorsiflexion/plantarflexion range of motion was −45° to +55°. Off-the-shelf shoes with modified soles were used for structural connection. The system was powered by a 24 V, 6000 mAh lithium-ion battery pack. The total mass of the device (including backpack) was approximately 5.1 kg. All validation experimental procedures were performed in accordance with the relevant guidelines and regulations, and written informed consent was obtained from the participant.

### 2.2. Human-Stylized Gait and Torque Prediction Algorithm

We implemented the AMP algorithm within the Isaac Lab. The human model was configured with anthropometric parameters corresponding to a male subject (height: 180 cm, mass: 70 kg). Segment lengths and geometries were derived from direct physical measurements, modeled in SolidWorks 2022 (Dassault Systèmes, Vélizy‑Villacoublay, France), and exported to URDF format using the SW2URDF plugin. IMU and laser ToF sensors were implemented as plugins in the simulation, with placements aligned to those on our ankle exoskeleton. Reference motion trajectories for the AMP policy were captured from the same participant using an optical motion capture system. The same reference trajectories were also used for the OpenSim inverse dynamics pipeline. After data preprocessing and cleaning, these trajectories were retargeted to the AMP agent following the official Isaac Lab documentation. We used the default configuration with domain randomization provided by Isaac Lab. To enable velocity command-following, an additional reward term penalizing deviations from target velocity commands was added. All simulation data were acquired directly via the native Isaac Lab interfaces.

The proposed algorithm, termed CNN-Transformer, employed a hybrid encoder–decoder architecture integrating feature extraction with attention-based sequence modeling. Input sensor data (8 channels) were first processed by a CNN encoder, which down-sampled the input and expanded the feature dimension to 128. The extracted features were then fed into a Transformer encoder of three identical layers. Each encoder layer utilized multi-head self-attention and a position-wise feed-forward network (hidden dimension 512), stabilized by layer normalization and dropout (*p* = 0.1). Subsequently, a Transformer decoder (three layers) refined these representations through masked self-attention and cross-attention mechanisms. Finally, decoded features were projected via a fully connected linear layer to a scalar torque output. The training process was configured to ensure robust convergence and generalization. Data were split into training, validation, and test sets at a ratio of 70:15:15. The loss function was combined with Mean Squared Error (MSE) for regression with L2 regularization. The Adam optimizer was used with an initial learning rate of 0.001 and a weight decay of 0.1. The learning rate was reduced by 50% every 50 epochs to support fine-tuning. We trained the model for a maximum of 500 epochs with a batch size of 32. Early stopping was implemented with a patience of 50 epochs, monitoring the validation loss.

### 2.3. Participants and Statistical Analyses

Participants and experimental protocol. The participant was an able-bodied adult male (age: 26 years, height: 180 cm, body mass: 80 kg, dominant limb: right). The participant was physically active and experienced with laboratory walking tasks. Written informed consent was provided. Each trial encompassed gait initiation, at least 10 consecutive gait cycles, and final termination (approximately 20 s per trial). Motion capture and GRF data were collected, time-synchronized with the exoskeleton sensor data. A bilateral lower extremity musculoskeletal model (OpenSim Gait2392) with 23 degrees of freedom, with a simplified torso, was used to compute ground-truth ankle joint moments. The participant completed two sequential roles in this study: powered exoskeleton (Exo on) and unpowered baseline (No Exo). A minimum 5 min rest period was provided between trials to avoid fatigue. No Exo trials were used for accuracy validation of the algorithm or to generate reference motion trajectories for AMP agent training on an instrumented treadmill. Data were collected at walking speeds of 0.6, 1, 1.25, 1.5, 1.75, 1.9 m·s^−1^, with approximately 20 trials per speed. Exo on trials with a controller were used to compute the mechanical ankle work on an instrumented walkway.

Statistical Analyses. Two standard metrics were used to quantify prediction accuracy: the root-mean-square error (RMSE) with standard deviation (SD), and the squared Pearson correlation coefficient (R^2^) with SD. The mathematical definition is: “$R^{2}=1-\frac{\sum_{i=1}^{n}(y_{i}-{\hat{y}}_{i}{)}^{2}}{\sum_{i=1}^{n}(y_{i}-\overset{-}{y}{)}^{2}}$ and $RMSE=\sqrt{\frac{1}{n}\sum_{i=1}^{n}{\left(y_{i}-\hat{y_{i}}\right)}^{2}}$, where $y_{i}$ was the ground truth, ${\hat{y}}_{i}$ was the predicted torque, and $\overset{-}{y}$ was the mean of the ground truth. RMSE represented the absolute metric of error relative to ground truth.” R^2^ provided a dimensionless goodness-of-fit metric that indicates the fraction of variance in the ground-truth ankle moments explained by the CNN-Transformer estimates. R^2^ also evaluates the similarity of moment curve shape, independent of amplitude scaling or constant bias.

Specifically, the CNN-Transformer model was trained to map contralateral swing-phase sensor data to the corresponding ipsilateral push-off torque profile, treating each gait cycle as an independent observation. The stride-level ensemble captured substantial variability from: (i) natural stride-to-stride fluctuations in kinematics, step length, and cadence; (ii) within-trial gait transitions (acceleration, steady-state, and deceleration); (iii) varying locomotor demands across the three target speeds. Thus, the relevant statistical unit was the individual gait cycle (stride), not the participant. Model performance was evaluated over large stride ensembles across locomotion conditions. To evaluate the effect of exoskeleton assistance on ankle positive mechanical work, stride-level values from the Exo on condition were compared to the No Exo baseline at each speed using a stride-level paired *t*-test (scipy.stats.ttest_rel, Python 3.11). Statistical significance was defined as *p* < 0.05. Reductions in user ankle positive mechanical work are expressed as absolute changes relative to the No Exo baseline within each speed condition.

## 3. Results and Discussion

**Figure 1:**
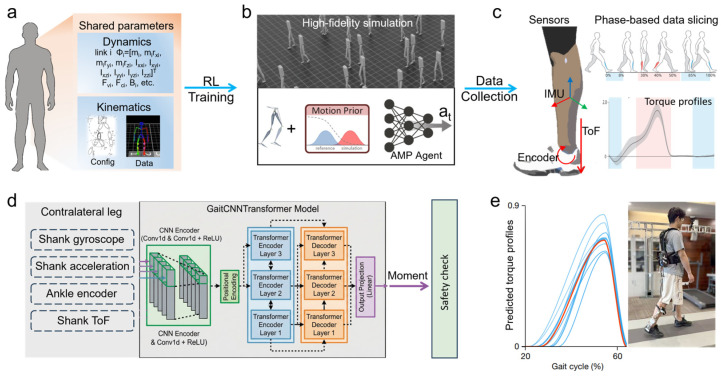
(**a**) Detailed kinematic and kinetic parameters based on human body for simulation training. (**b**) High-fidelity Isaac Sim as parallel RL training environment with AMP agent to imitate human-like gait at variable walking speeds. (**c**) Sensor inputs comprised IMU, laser ToF, and encoder data, with specific mounting positions. Gait cycle was segmented, and ankle biomechanical torque profiles were obtained through OpenSim. (**d**) CNN-Transformer algorithm with encoder–decoder architecture, taking 8-dimensional data as input and safety check before motor execution (**e**).

This work leveraged advanced imitation learning and physics-based simulation environments to facilitate a paradigm shift from complex dynamic models to data-driven biomechanical estimation. An algorithmic framework was established based on the principles of human bilateral coordinated locomotion. First, detailed kinematic and kinetic parameters were generated based on the human body for model files in simulation training (Figure 1a). The kinetic data for link *i* comprised the inertial parameter vector **Φ_i_** = [m_i_, m_i_r_xi_, m_i_r_yi_, m_i_r_zi_, I_xxi_, I_xyi_, I_xzi_, I_yyi_, I_yzi_, I_zzi_]^T^, viscous friction coefficients **F_vi_**, Coulomb friction coefficients **F_ci_**, motor inertia parameters **B_i_**, etc. The kinematic data included the kinematic coordinate and lower-limb gait data acquired through optical motion capture. The upper extremities simplified as a single rigid body. The collected data also served as a reference motion for AMP training. A massively parallel physical simulation concurrently ran 4096 agents for training. The AMP framework leveraged a discriminator to distinguish between generated and reference motion distributions, thereby incentivizing the policy to produce behaviors that closely match the reference data distribution (Figure 1b). This adversarial training objective ultimately yields an AMP policy capable of human-like locomotion (Video S1). The AMP policy could also generate specific pathological gait patterns by utilizing corresponding motion data. To mitigate overfitting, early stopping was employed based on validation performance.

The sensor configuration is illustrated in Figure 1c. Sensor inputs comprise IMU, laser ToF, and encoder data. Identical sensor types and mounting positions were adopted across simulation and experimental platforms, ensuring data fidelity and facilitating sim-to-real transfer. Gait cycle onset was defined by heel strike. In simulation, gait phase detection was implemented using plantar force sensors at the heels and metatarsal heads. For experimental deployment, phase-detection used laser ToF to identify heel strike via a calibrated threshold. The mean timing offset against motion capture was 12.4 ± 2.1 ms, which is negligible for gait phase control. As shown in Figure 1d, the proposed torque profile generation algorithm adopted an encoder–decoder architecture, taking 8-dimensional data as input. CNN performed feature extraction on the input data, while Transformers were employed to predict the torque sequence. The self-attention mechanism inherently enabled the generation of variable-length output sequences across varying gait cycle durations. This non-real-time generation was adopted for two considerations: First, computational cost on embedded hardware was minimized. Second, a pre-deployment safety check was enabled, allowing validation against predefined biomechanical constraints prior to actuation. The safety check layer imposed hard constraints on the cycle period and peak torque clipping. Such precautionary measures were deemed essential for exoskeleton applications. Finally, the proposed torque prediction algorithm was deployed on a custom ankle exoskeleton for experimental validation. The system demonstrated robust generalization across variable walking velocities, with generated torque profiles exhibiting satisfactory tracking performance and biomechanical fidelity.

**Figure 2:**
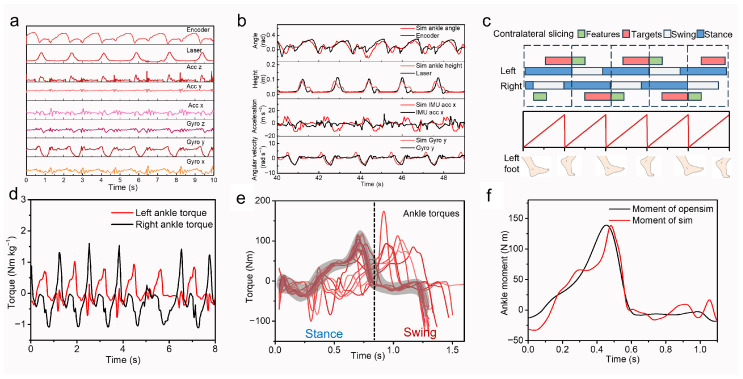
(**a**) Experimental sensor data of a walking velocity of 0.6 m·s^−1^. (**b**) Sensor data comparison between simulation and experiment at 0.6 m·s^−1^. (**c**) Dataset construction workflow. Contralateral data were segmented in the footfall pattern, where contralateral swing phase (green) was utilized to predict stance phase torque profiles (red). (**d**) Torque profiles of ankle dorsiflexion/plantarflexion in simulation. After segmentation, simulation showed high-fidelity stochastic variations in torque profiles (**e**). (**f**) Comparison between our simulation and standard experimental OpenSim pipeline.

Experimental data were acquired using the custom ankle exoskeleton, encompassing 8 sensor channels, joint angle encoders, ToF sensors, and IMUs, capturing triaxial acceleration and angular velocities, as shown in Figure 2a. The gyro y corresponded to the sagittal plane of the human body, the laser ToF distance corresponded to foot heel clearance from the ground, and the encoder corresponded to joint angular displacement. With the trained AMP agent of human-like gait in a simulation environment, we cross-validated the above 4 important channels data with experimental measurements. As shown in Figure 2b, the simulation not only preserved the characteristics but also captured physically consistent details, such as subtle peaks in the laser sensor signal, etc. Figure 2c shows our dataset construction workflow; the kinematic data of the contralateral swing phase (green) were employed as the input features for the CNN-Transformer. The upcoming stance-phase push-off torque of the ankle joint (red) was collected as the target curve, as shown in the foot fall pattern in Figure 2c. The heel strike event was utilized as the temporal onset for each gait cycle. Figure 2d showed the obtained ankle torque profiles, normalized to 70 kg body mass. The prominent positive peaks in the torque curves corresponded to the active push-off phase, providing propulsive force to walk forward. We noted that discernible bilateral asymmetry and inter-cycle variability existed, inheriting the stochasticity of the Isaac Sim physics engine. Rather than exhibiting rigid periodicity, the gait remained quasi-periodic, with no two cycles being identical. This characteristic ensured that the synthesized gait more closely approximated real-world human locomotion, where physiological fluctuations and real-world environmental perturbations prevented exact cycle-to-cycle replication.

We collected about 50,000 trials, and each encompassed a complete gait, including standing initiation, ten steps of steady-state walking, and final termination. Figure 2e presents the selected samples from the dataset. Joint torques exhibited consistency during steady-state walking, whereas minor deviations were observed in a subset of data corresponding to acceleration and deceleration phases. By deploying 4096 agents in parallel, we amassed substantial data within a few minutes. Besides enhanced realism and scalability, our simulation-based data generation strategy was rigorously validated against the conventional gold standard pipeline. Typically, optical motion capture for kinematics and force plates for ground reaction forces (GRFs) were used to collect data. OpenSim with inverse dynamic pipelines was used to calculate ground-truth joint torques. The pipeline was to solve an optimal control problem with a set of costs under boundaries, rigid body dynamics, and muscle dynamics constraints. This work does not involve muscle characteristics, so the rigid body dynamics were important, which are often defined as: $M\left(q\right)\dot{u}+{G\left(q\right)}^{T}\lambda =f_{app}\left(t\right)-f_{inertial}\left(q,u\right)$, where specific symbols can be referenced to [21]. We compared representative joint torque profiles from the AMP agent against OpenSim output, as shown in Figure 2f. Given that both methods adopted the same motion capture data as input, the comparison was directly valid. The AMP-derived profiles exhibited richer high-frequency details across both stance and swing phases. We attributed the smoother OpenSim curves to the aggressive filter required for inverse dynamics convergence, an artifact avoided by our proposed approach.

**Figure 3:**
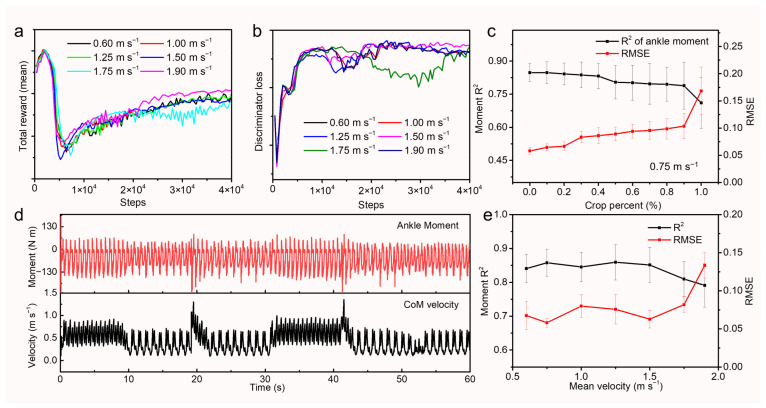
(**a**) At varied velocities (0.6–1.9 m·s^−1^), AMP agents exhibited consistent policy performance with converged training rewards. (**b**) Discriminator losses of AMP agent at varied walking speeds. (**c**) Relationship between swing phase data cropping and prediction accuracy at 0.75 m·s^−1^. (**d**) CoM velocity and joint torque throughout walk episode. (**e**) RMSE and R-square characteristics of predicted torque profiles.

Walking velocities in real-world scenarios exhibited significant variability, both between different trials and between individual steps. Consequently, we conducted a comprehensive evaluation to quantify the impact of velocity variations. Varied walking speed ranging from slow 0.6 m·s^−1^ to very fast 1.9 m·s^−1^ was studied. Additional reward terms were added to the total reward during training to endow velocity-tracking capability. As shown in Figure 3a, the reward curves exhibited convergence, indicating that the policies effectively learned walking skills against speed variations. To prevent the erosion of human-like gait characteristics during high-velocity training, we increased the weights of the style reward, which penalized deviations from the reference dataset’s behavioral style. This strategy ensured that the discriminator loss converged to comparable levels across all tested velocities (Figure 3b). Reasonably, high walking velocities had shorter swing phases and posed a challenge for temporal feature extraction. By cropping swing phases data fragments from back to front of 0.75 m·s^−1^, we conducted an ablation study, and the result is shown in Figure 3c. From 0.6 m·s^−1^ to 1.9 m·s^−1^, RMSE increased from 0.058 ± 0.006 Nm kg^−1^ to 0.168 ± 0.044 Nm kg^−1^ while R^2^ decreased from 0.848 ± 0.041 to 0.711 ± 0.116. The model maintained high accuracy with up to the last 40% swing phase data filled with 0. Conversely, when the input data was entirely absent (100% truncation), the algorithm defaulted to outputting the expectation of all reference curves. Figure 3d showed a representative walking trial, including the gait initiation, steady-state walking, and variable velocities, characterized by intermittent acceleration and deceleration. By directly extracting joint torque data, we observed that torques were minimal during slow walking but increased significantly during acceleration phases. Concurrently, the sagittal plane velocity of the center of mass (CoM) was synchronously recorded to correlate with these dynamic torque variations, as shown in Figure 3d. We segmented joint torque profiles specifically within the push-off phase across complete gait cycles to evaluate prediction accuracy. The algorithm demonstrated robust performance across the velocities, maintaining high fidelity from slow to high speeds as shown in Figure 3e. From 0.6 m·s^−1^ to 1.9 m·s^−1^, RMSE increased from 0.067 ± 0.018 Nm kg^−1^ to 0.13 ± 0.016 Nm kg^−1^, while R^2^ decreased from 0.84 ± 0.04 to 0.79 ± 0.06. A degradation in accuracy was observed at 1.75 m·s^−1^. This decline was likely attributable to insufficient effective data points caused by the interaction between rapid gait dynamics and the sensor system’s fixed sampling frequency (50 Hz), thereby limiting temporal resolution during fast locomotion.

**Figure 4:**
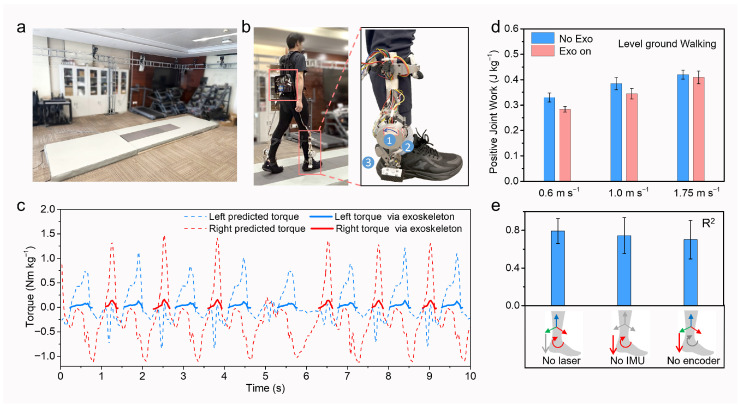
(**a**) Experimental setup comprised an optical motion capture and an instrumented walkway with force plates. (**b**) Exoskeleton device utilized for CNN-Transformer algorithm deployment, with integrated sensor placements aligned in simulation: (1) IMU, (2) ToF, and (3) encoder. (**c**) Predicted bilateral ankle joint torque profiles and corresponding assistive torque delivered by exoskeleton during variable-speed walking. (**d**) Reduction in human mechanical work attributable to exoskeleton assistance. (**e**) Variation in accuracy under conditions of partial sensor failure within our multi-sensor system.

Subsequently, we deployed and validated the proposed CNN-Transformer prediction algorithm on our exoskeleton platform. The validation protocol involved variable-speed level walking on a footpath with a force plate and optical motion capture, as shown in Figure 4a. The exoskeleton and sensor configuration were detailed in Figure 4b. Notably, the system employed a single motor to provide assistive torque at the ankle joint. Each experimental trial encompassed a comprehensive locomotion sequence: gait initiation, steady-state walking, intermittent acceleration, deceleration, and final termination. As illustrated in the representative data segment (Figure 4c), the joint torque profiles exhibited distinct variations corresponding to these dynamic gait transitions. The exoskeleton, with intrinsic threshold phase detection, delivered only plantarflexion torque during the push-off phase, as depicted by the red solid line in Figure 4c. Specifically, upon detection of the torque activation, the system executed proportional assistance based on the predicted torque profile. This fixed percentage of joint moment method is derived from the proportional joint-moment control (PJMC) method [38]. Given the motor’s torque limits, the assistance gain was set to 10% of the predicted profiles. The total mechanical work of the joint was obtained by inverse biomechanical calculations. The interaction torques were obtained from motor current feedback. The 100% baseline of positive joint work was defined as the condition in which participants were not wearing the exoskeleton.

To analyze the effect of our controller at the joint level, mechanical work was measured under two conditions: with and without the powered exoskeleton. As shown in Figure 4d, the total positive mechanical work of the user’s ankle joints (the sagittal plane) was significantly lowered with the powered exoskeleton compared to in the no-exoskeleton condition during level-ground walking [change of 0.046 ± 0.021 J kg^−1^ at 0.6 m·s^−1^, 0.039 ± 0.032 J·kg^−1^ at 1.0 m·s^−1^, and 0.011 J·kg^−1^ at 0.030 m·s^−1^]. Given the use of multiple sensors in our setup, we investigated the performance of fault-tolerant ability, a critical consideration for safety in multi-sensor systems. By individually disabling each sensor module and applying corresponding dropout during training, we observed no significant degradation in accuracy (Figure 4e). Notably, the encoder was found to play a vital role in torque estimation; its absence led to a substantial decline in accuracy (29.8 ± 17%), suggesting that the encoder provided higher data quality and was more correlated with torque computation. During the human push-off phase, all three sensors generated responsive signals: the encoder directly captured joint angles, the IMU tracked the shank’s pose, and the laser monitored foot–ground interaction. This demonstrates that our sensor system configuration possesses sufficient redundancy, thereby ensuring safety.

## 4. Conclusions

This study established a unified, synthetic data-driven framework for exoskeletons that effectively generalized across a broad range of walking velocities. Using massively parallel physics-based simulation, we generated high-fidelity biomechanical datasets from AMP agents, without labor-intensive artifacts filtering or conventional OpenSim inverse dynamics pipelines. We proposed a CNN-Transformer architecture, which leveraged contralateral swing-phase sensor data to predict variable-length push-off torque profiles, demonstrating robust prediction accuracy and seamless sim-to-real transferability. Experimental validation confirmed that this controller significantly reduced user ankle positive mechanical work, ranging from 0.6 m·s^−1^ to 1.75 m·s^−1^. And the controller significantly reduced user ankle positive mechanical work by up to 14 ± 6.30%. Furthermore, our multi-sensor configuration exhibited inherent fault tolerance; while encoder data proved most critical for precision, the complementary information from IMU and ToF sensors ensured safe, stable operation even under partial sensor failure. This study has limitations regarding generalizability, as it did not involve demographic participants. The experiments, as an algorithmic demonstration of the concept, were validated primarily on flat terrain, and physiological impacts, like metabolic rate, were not fully measured. The primary significance of this work lies in the successful validation of the synthetic data-driven paradigm. Future work will address these gaps to validate the clinical significance. Meanwhile, this synthetic data-driven paradigm offers a scalable foundation for developing controllers capable of managing complex locomotor tasks and multi-joint coordinated assistance. Ultimately, this work demonstrates that scalable, data-driven approaches can replace rigid, handcrafted control strategies, offering a practical and adaptable pathway toward deploying data-driven exoskeletons in unconstrained, real-world environments.

## Data Availability

All produced data are available within the manuscript.

## Supplementary Materials

The following supporting information can be downloaded at: https://www.mdpi.com/article/10.3390/biomimetics11050319/s1. Video S1: Diverse variable-speed walking scenarios in simulation used for data collection.
